# Book Reviews

**Published:** 1913-12

**Authors:** 


### BOOK REVIEWS

*Books mentioned may be inspected at and ordered through this office. So far as possible, books received in any month will be reviewed in the issue of the second month following.*

DIAGNOSIS OF THE MALIGNANT TUMORS OF THE ABDOMINAL VISCERA. Rudolph Schmidt, Professor of Medicine, University of Innsbruck. Authorized English version by Joseph Burke, Sc. D., M. D., attending surgeon Buffalo Hospital of the Sisters of Charity, Consulting Surgeon Emergency Hospital, Buffalo, N. Y. Rebman Co., Herald Square Building, New York City. Cloth, $4.00.

To those members of the medical profession who are unfamiliar with the German language, this English version of Professor Schmidt's work is particularly welcome. The occult path ology of the abdominal cavity in its protean and mysterious phases is herein brought under the calcium and focussed into a state of comparative lucidity. Besides numerous original and trustworthy signs and tests, especial stress is placed upon the logical evolution of the patient's story in differentiating between incipient carcinoma (an immediate problem for the surgeon) and various benign and symptomatic pathologies which belong distinctly to the domain of the internist. As Schmidt so aptly says: "To choose the right path between these two extremes of possible error belongs to the most difficult problems of internal medicine."

Each chapter emphasizes the import of its predecessor, but the one on "Case Histories" is absorbing in its interest—the analogue of Murphy's surgical clinics. To the American who has stood at the bedside in the Allgemeines Krankenhaus and listened to the analysis of an obscure abdominal lesion by Schmidt, no word expresses this clinician's acumen and intuition better than that of the translator "uncanny."

The "uebersetzung"—unlike many others—holds all the spirit and force of the original.

Dr. Burke, by the three years of intimate association in his earlier practice with Neusser and Schmidt, has absorbed the ratio-cination habit from these past-masters, and he retains and manifests Schmidt's deductive principles throughout this most interesting translation.

Of added importance in this connection is the fact that Dr Burke has—for the past ten years—been substantiating the anticipated findings of Schmidt as outlined in his "suspicious factors and differential diagnosis" by his own counter findings in an extensive surgical clinic.

This book addresses itself most directly to the physician who, by the early recognition of malignancy, would add years to the span of human life.

---

Applied Pathology in Diagnosis and Treatment. Julius M. Bernstein, M. B., D. P. H., M. R. C. P., London published by the University of London Press, represented by American Branch of Oxford University Press, 35 W. 32, New York; 395 pages, 5 colored plates, 46 drawings; $3.75.

This is a very useful work dealing with microscopy, bacteriology, many of the modern serum reactions, diagnostic and therapeutic and the like.

---

Medical Diagnosis—A Practical Treatise for Students and Practitioners, by the late John H. Musser, M. D., LL. D., of Philadelphia, sixth edition revised by John H. Musser, Jr., B. S., M. D.; published by Lea & Febiger, Philadelphia and New York; 793 pages, 196 engravings and 27 colored plates.

Having personally followed the teaching of the late Dr. Musser, this work is a reminder of pleasant days spent in Philadelphia. His son, while ably conducting the work of revision necessary for every text book in these days of rapid advancement of medical science, has preserved the original text so far as possible. The book begins with a general discussion of diagnostic methods, including history taking. The second section covers various symptoms, such as pain, dysphagia, diarrhœa, speech, etc. The third section of 130 pages deals with what is technically termed inspection and some other superficial methods, well grouped under the term "observation." We call especial attention to this section, since it recognizes the value of older methods of detection of disease and tends to inculcate that art of medical diagnosis, which we admire in the great men of medical history and which we are so prone to neglect at present. Sections on physical and laboratory diagnosis follow. Part II, Special Diagnosis, is classified according to Infestions, diseases due to Animal Parasites, Intoxications, Metabolic Diseases, and the various systems and organs.

---

Anatomy, Descriptive and Applied, by Henry Gray, F. R. S., New English Edition, with the Basle Anatomic Nomenclature in English, by Henry Howden, M. A., M. B., C. M., Professor of Anatomy in the University of Durham, Eng. Published by Lea & Febiger, Philadelphia and New York; 1407 pages, 1126 engravings; $6.00 cloth, $7.00 leather.

In the October issue we reviewed the corresponding American edition. The difference between the two can best be given in Prof. Howden's words: "The outstanding modification in the text of this edition is the use of the Basle nomenclature . . . . . Where the Basle nomenclature differs materially from the older terminology, the latter has been added in brackets and for further convenience, a glossary is appended showing (a) the terms adopted in the text, (b) the Basle, and (c) the old terminology." The paragraphs on surface anatomy have been placed together to form a separate chapter, and the section on histology has been shortened by describing the complex tissues along with the organs to which they relate. Some differences in the new illustrations occur also. We note the general adoption of metric units, the absence of the u from color, and, indeed, the only expression that suggests that this is an English edition, is the author's use of the word "outstanding" in the passage quoted from the preface, in a sense quite obvious and correct but very different from that in which Americans usually employ the word. Like the corresponding American edition, this work is a magnificent modernization of a classic medical book and we are at a loss to choose between them.

---

Mortality Statistics, 1910. Eleventh Annual Report of the Bureau of the Census, Dept. of Agriculture, Wm. J. Harris, Director, prepared under the supervision of Cressy L. Wilbur, M. D., Chief Statistician for Vital Statistics.

This volume, in which mortality statistics can be directly compared with those of population, without estimating the latter, is especially satisfactory as a basis of various studies. Perhaps on this account the mortality rate (15:1000 population) is slightly higher than for the preceding two years but lower by 1.2 than in the average for 1901-5. The average age at death is 38.1 for males, 39.4 for females, general average 38.7, as compared with 35.2 for 1900. No significant difference in conditions occurs to us to modify the conclusion that hygienic and sanitary methods have really prolonged human life by an average of 3½ years in the last decade. The alarm over the increase of cancer so generally expressed, ought to be mitigated by the fact that the average age of death is 53.8 for cancer of the female genitals and from 57.7 to 70 for other specified locations. The same general explanation is more or less applicable to other causes of death that have shown a marked increase in the last generation, namely, that a greater number of the population live to a vulnerable age.

---

University of the State of New York Bulletin. Museum Bulletin No. 164, Ninth Annual Report of the Director of the Science Division, published in book form at Albany by the State Education Department.

This volume contains a general description of the museum and its educational work and reports in geology, botany, entomology, soology, archæology, etc. In the last we note an illustrated description of a beautiful collection of slate knives, of Eskimo origin, found at Glen Lake and donated to the museum by Dr. Albert Vander Veer of Albany.

---

Essentials of Prescription Writing. By Cary Eggleston, M. D., Instructor in Pharmacology, Cornell University Medical College, New York City. 32 mo. of 115 pages; W. B. Saunders Company, 1913; cloth, $1.00 net.

This is a very convenient little book of pocket size, containing the usual information, well classified. Just as an illustration of the fact that experienced physicians, as well as medical students, need this work, we acknowledge with due humility that we have just learned from it that we ought to have written "oleum theobromatis" instead of oleum theobromæ, for the past twenty-five years.

---

A Clinical Manual of Mental Diseases. By Francis X. Dercum, M. D., Ph. D., Professor of Nervous and Mental Diseases, Jefferson Medical College, Philadelphia. Octavo of 425 pages. Philadelphia and London: W. B. Saunders Company, 1913; cloth, $3.00 net.

We welcome this work from the pen of one of the best teachers that we ever had. It is based on the author's lecture course, and, while simple, is thorough. The eminently sane viewpoint of the author is well shown by this quotation from the preface: "In view of the recrudescence within recent years of speculative and metaphysical psychiatry, it may seem an act of temerity to present the subject from the clinical point of view . . . . ." It is well that someone has had this temerity, and no one is better prepared than the author to discuss insanity as a diseased state, requiring the services of a physician and not a psychologist.

---

Pocket Cyclopedia of Medicine and Surgery, Gould & Pyle, second edition, revised and enlarged by R. J. E. Scott, M. A., B. C. L., M. D., published by P. Blakiston, Son & Co., Philadelphia; illustrated; $1.00.

The masterly skill of George M. Gould as a lexicographer and compiler was ably seconded by Dr. Pyle and Dr. Scott, has caught the spirit of the original authors, and, while bringing the work up to modern requirements, he has not spoiled it by too much editing. The work is a good deal more than a dictionary. It contains tables and illustrations that condense an almost incredible amount of technical knowledge in small compass, while the publisher's art has reduced the weight of paper to a minimum without sacrificing clearness of print. While the present work makes no pretense of supplying the demands of a reference library, it is just what is needed as a pocket companion, particularly in the trying early years of practice when the physician so often wishes to refresh his memory, and, without reference to experience in traveling when baggage must be condensed.

---

Diseases of the Skin. Including the Ex-Anthemata, for use of General Practitioners and advanced Students. By Frederick M. Dearborn, A. B., M. D., Professor of Dermatology in the New York Homopathic Medical College and Flower Hospital. 230 illustrations in the text; 551 large 8 vo. pages; cloth, $5.00 net; postage, 30 cents. Philadelphia: Boericke & Tafel, 1913.

The author first discusses the histology of the skin and its appendages, then gives a systematic description of skin lesions in general, of etiologic factors, methods of diagnosis, classification, etc. The systematic discussion of individual diseases follows with illustrations. So far as external treatment and the use of imponderable agents are concerned, the author's homeopathic affiliations do not seem to be of influence and the indications for internal treatment are stated in very general terms, without dosage or specification of preparations. Under the general category of symtomatic treatment, he gives a considerable list of homeopathic remedies with potencies to be used, and, after each drug, the names of the diseases in which it is applicable. This impresses us as indicating a commendable loyalty to his own school of practice, joined with a broad view of the general problems of therapeutics.

---

The Practical Medicine Series, Vol. 6, General Medicine; 355 pages; $1.50. The entire series of ten volumes is sold for $10.00. The general editor is Charles L. Mix, A. M., M. D.; the editors of this volume are Frank Billings, M. S., M. D., and J. H. Salisbury, A. M., M. D., all of Chicago. Published by the Year Book Publishers, Chicago.

The wide range of subjects covered in a review of current literature, renders it difficult to select any particular point for discussion. However, the present volume deals mainly with infections and gastro-enterology. Both for reference and in reviewing rapidly the advances in these branches of medicine, the volume is very useful. We strongly advise, however, the purchase of the entire series.

---

Obstetrics. A Manual for Students and Practitioners. By W. P. Manton, M. D., Professor of Obstetrics and Clinical Gynecology, Detroit College of Medicine, Detroit, Mich. Second edition, revised and enlarged; including selected list of State Board Examination Questions. 12mo, 292 pages, with 97 engravings. Cloth, $1.00 net. Lea & Febiger, Publishers, Philadelphia and New York, 1913.

This is one of the red-cloth, white-lettered, Medical Epitome Series. While avowedly prepared mainly for preparation for examinations, the subject is treated in a descriptive manner, each chapter being followed by a series of unanswered questions, which will suggest to the student whether he has mastered that part of the subject. This arrangement imposes a sufficient amount of labor to impress forgotten points upon the memory, without requiring one to turn back over the entire book. This is a fair compromise between the question and answer method of the usual quiz compend and the purely didactic text book. It should not be forgotten that, even with no examination in prospect, it is a good plan for the practicing physician to brush up occasionally, on an entire subject, getting a bird's eye view of it. Journal literature necessarily covers the field of medicine unsystematically and deals mainly with new or controversial topics. On the other hand, more ambitious medical books tend to deal more and more exhaustively with some single subject. Epitomes fix in the mind the general features of a subject, and, so to speak, give a basis of orientation for the details acquired from more highly specialized discussions.

---

Medical Directory of New York, New Jersey and Connecticut, published by the Medical Society of the State of New York. Vol. 15, issued September 20, 1913. The publication committee has, with its usual modesty, announced no names and it asks for corrections, not for words of appreciation. But every member of the Society ought to feel grateful for the great care bestowed on this work.

17,958 physicians are listed—13,777 in New York, 2,685 in New Jersey, 1,396 in Connecticut, representing an increase of 81, 11 and 2 for the respective States. The physicians of Greater New York City number 7,427, a gain of 62, those of the rest of the State, 6,350, a gain of 19. These increases are insignificant in comparison with a gain in population for the State of New York of about 180,000 a year, but the report is not so favorable as last year, when an actual decrease was noted.

For Buffalo, the following statistics may be of interest. The 1912 directory listed 703 names, to which four were added and from which 28 were subtracted, immediately—net 679. The present directory lists 698 names, to which one is to be added and from which 36 are to be subtracted—net 663. Twenty-four new names occur in the present list: 6 of internes in hospitals, 17 of new practitioners and 1 of a local office by a suburban resident. The subtractions mentioned include one recently deceased and two known to have moved from the city, and medical graduates who are retired or who are engaged in other occupations, as teaching, dentistry, pharmacy and, in the case of several ladies, managing other physicians and their homes.

We propose a slogan for the medical profession of Buffalo—500,000 people, 500 doctors—but we make this suggestion with an assurance of peaceable intentions.

The number of physicians who reform is surprising. In one day we recently met, socially or on business, four men still young, who had abandoned practice. Every little while we are surprised to learn that some business acquaintance has a dust-covered medical diploma.

---

Proceedings of the Royal Society of Medicine, Vol. 6, No. 9, July 1913. Published by Longmans, Green & Co., London and New York. Price, 7/6d.

The various section meetings are reproduced, the multiplicity of topics rendering a review difficult. The general consideration of Balneology and Climalogy impresses us as the part of the work which will afford the most new light for our readers.

A Journey to the Earth's Interior or Have the Poles Really Been Discovered? Marshall B. Gardner, Aurora, Ill. Published by the author.

The author holds the theory of a sun within the interior of the earth, with continents and water on the inner surface of the hollow sphere constituting the earth. He believes that the concavity and the convexity of the earth are united at the poles, and that the open polar sea, northward migration of arctic birds and mammals, finding of mastodons frozen in the ice, etc., indicate an open communication at the poles. The dip of the magnetic needle is similarly explained. The polar pit or polar opening has been exploited in fiction by several authors in delightful stories. Mr. Gardner's hypothesis is so alluring in many ways, practical as well as theoretic, that we are inclined to express the hope that the discoveries of the poles will prove incorrect. It occurs to us, however, that there are some hard facts in regard to gravitation that may interfere with the realization of this hope, even if we grant the liability to error of observations on latitude taken by the reputed discoverers of the poles.

---

Hand Book of the Mental Hygiene Movement and Exhibit. Published by the National Committee for mental hygiene, 50 Union Square, N. Y.; 25 pages, 31 charts; 25 cents, postpaid.

This is an interesting pamphlet, containing statistic tables, diagrams of adjustment, illustrations of lesions and information as etiology.

---

Indigestion, Constipation and Liver Disorder. G. Sherman Bigg, F. R. C. S., etc., London; 168 pages; $1.50; published by Paul B. Hoeber, 69 E. 59, N. Y.

Very briefly, the physiology of digestion and the various physical methods of investigation are described. We are pleased to note the praise bestowed on auscultatory percussion. It is scarcely "an original idea" (see essay by editor in Transactions of the Medical Society of the State of New York, 1905), but has been used more or less for half a century. While, as advised, the stomach—or other organ—may be mapped out by "gently gliding the ear piece of the stethoscope over the surface," it is usually more satisfactory to hold the ear piece stationary over the part of the organ most directly in contact with the body wall and to move the percussing finger (or tuning fork, etc.) "It is possible . . . . . to mark out the exact extent of a dilated stomach with even greater accuracy than that afforded by Röntgen rays." While admittedly an enthusiast over auscultatory percussion and allied methods of detecting visceral transsonance, we think this statement requires some qualification. We were the first in America to locate the stomach by bismuth and Röntgen rays (being a few months later than Roux in publishing but acting independently) and, as the results coincided closely with those previously obtained by auscultatory percussion, we have usually adhered to the latter method, which is more convenient and devoid of danger. A good radiograph gives a more exact delineation of a stomach than auscultatory percussion and fluoroscopy with a clear screen, in favorable cases, throws light on peristalsis which cannot be obtained so strikingly by any acoustic method. But, unless one bears in mind that the radiograph is an instantaneous picture, showing marked indentations due to peristalsis, that the bismuth at any given moment does not necessarily fill the entire stomach, and makes other allowances for the bizarre shapes into which the stomach throws the bismuth, he will not get so good an idea of the general shape and size of the stomach as from auscultatory percussion, and he will diagnose hour-glass contraction, up-right stomach and various other abnormalities from purely transient distortions of a normal stomach.

Under the head of Acidity, we fail to find either methods of determining between fermentation and secretory superacidity or the sharp discrimination of therapeutics necessary.

These criticisms give a fair idea of the book in general. It is not closely analytic nor scientific in the modern sense, yet it contains many valuable suggestions for symptomatic treatment and is a good old-fashioned clinical monograph.

---

Diagnosis of Bacteria and Blood Parasites. E. P. Minett, M. D., etc., Georgetown, British Guiana, published by Paul B. Hoeber, 69 E. 59, N. Y.; 80 pages; $1.00.

This little book gives a very ingenious analytic system of examining pure cultures for the identification of bacteria; pathogenic and non-pathogenic; a general description of staining methods, and a discussion of special tests as the Wassermann, Widal, Calmette and other biologic reactions. Standardization of emulsions, methods of counting, blood culture, sterilization, post mortem examination of inoculated animals and allied subjects are then treated. The spirochæte pallida, piroplasma, Leischmann-Donovan bodies of kala-azar, malaria parasites, etc., are also described.

RADIUM, AS EMPLOYED IN THE TREATMENT OF CANCER, ANGIOMA, KELOID, LOCAL TUBERCULOSIS, Etc. Louis Wickham, M. V. O., and Paul DeGrais, Paris, translated by A. and A. G. Bateman, M. B., C. M., published by Paul B. Hoeber, 69 E. 59, N. Y.; 111 pages, illustrated; $1.25.

A brief description of radium and radio-activity is given. It is stated that naked radium emits 90 per cent. of alpha rays, 9 per cent. of beta rays and 1 per cent. of gamma rays. Owing to the differences of penetration of the glass wall, the emanations as practically employed consist of 7, 90 and 2 per cent., respectively. "Cross fire" is especially considered and the appliances, reactions on tissues, etc., are described in a very practical way. It is perhaps a coincidence that the authors are ex-chiefs of the clinic and of the laboratory, respectively, of the St. Louis Hospital; and that their method of discussing the therapeutic value of radium is of the "show me" kind. The evidence submitted is most convincingly supported by photographs, though contra-indications and limitations are candidly admitted.

---

MODERN PROBLEMS OF BIOLOGY, Charles Sedgwick Minot, M. D., Boston, a series of lectures delivered at the University of Jena, December, 1912; published by P. Blakiston's Son & Co., Philadelphia; 124 pages; $1.25; 53 illustrations.

We need not comment on the well deserved honor conferred on Dr. Minot in the invitation to deliver these lectures. They cover: The New Cell Doctrine, Cytomorphosis, The Doctrine of Immortality, The Development of Death, The Determination of Sex and the Notion of Life. The first two lectures deal with technical knowledge, which is rather familiar to most of our readers and scarcely susceptible of review. The lecture on Immortality will prove disappointing to those who anticipate a religious controversy, as it discusses general methods of propagation, the improbability of spontaneous generation after the original development of life, polyembryony, etc. The development of Death is a scholarly discussion of phenomena of growth and of senescence. Osler's dictum that " a man is as old as his arteries," is quoted with approval. Metschnikoff's theory of the prolongation of life by the use of sour milk is met with C. A. Herter's experiments tending to show that lactic acid fermentation has no influence on the intestinal flora. No definite conclusion is reached as to the ultimate cause or nature of death. Analogously, the Determination of sex is not discussed with reference to crude notions of influencing the sex of offspring, but with reference to the law of chance in the propagation of different animals and to the general argument between intrinsic cellular causes and those due to environment.

CANCER OF THE BREAST, Charles Barrett Lockwood, F. R. C. S., London. Published by Henry Frowde and Hodder & Stoughton, represented by the American Branch of the Oxford University Press, 35 W. 32, New York City; 234 pages, illustrated; $3.00.

This monograph discusses clinical manifestations, the pathology of cancer and other neoplasms from which it must be differentiated and the local and complete operations, management of recurrent tumors and various matters suggested by a thorough consideration of the general subject. We miss the elaborate illustrations and detailed description of technic that would undoubtedly have been presented in an American monograph on the same subject, but, on the other hand, the general principles concerned are presented more clearly and more judicially and what may be termed permanent knowledge of this phase of practice is not obscured by hobbies of the moment. On the whole, we are inclined to think that while the English method here illustrated does not, at first sight, impress one so overwhelmingly with the erudition of the author and the enterprise of the publisher, the practical value of the book is greater.

---

PATHOLOGY, GENERAL AND SPECIAL. A manual for Students and Practitioners. By John Stenhouse, M. A., B. Sc. (Edin.) M. B. (Tor.), formerly demonstrator of Pathology, University of Toronto, Toronto, Canada. Second edition, revised and enlarged; including selected list of State Board Examination Questions. 12mo, 278 pages, illustrated; cloth, $1.00, net; Lea & Febiger, Publishers, Philadelphia and New York, 1913.

This is one of the red-cloth, epitome series, ably prepared to assist either the undergraduate or the graduate student confronted with an examination, and also, to enable the practitioner to obtain with little effort a bird's-eye view of the subject and a refreshing of his memory.

---

THE SAWYER SANATORIUM, White Oaks Farm, Marion, Ohio.

This is an elaborate descriptive pamphlet, illustrated so as to present the equipment of the institution for the treatment of Chronic nervous and mental diseases and the treatment and education of deficient and handicapped children.

---

PHYSICIANS' VISITING LISTS, P. Blakiston's Son & Co., Philadelphia.

These are arranged with a folding cover, envelope and pencil, containing various tables of doses, poisons, etc. Different forms are provided, for from 25 to 100 patients a day or week, at prices from $1.25 up. These lists are old favorites and need no introduction.

---

A COMPOUND OF DISEASES OF THE SKIN, Jay F. Schamberg, A. M., M. D., Philadelphia. Published by P. Blakiston's Son & Co., Philadelphia; 302 pages, illustrated; $1.25.

This is the fifth revised edition of the familiar, brown-cloth, quiz compend, long a favorite.

---

FIRST BOOK OF HEALTH, a text book of personal hygiene for pupils in the lower grades, by Carl Hartman, B. A., M. D., and Lewis Bradley Bibb, B. A., M. D., Austin, Texas. Published by the World Book Co., Yonkers; 155 pages, illustrated; 35 cents.

---

THE HUMAN BODY AND ITS ENEMIES, idem, 358 pages, illustrated; 65 cents.

The purpose of these two books, the latter entering into many more details of physiology and sanitation than the former, is, obviously, to educate the child of from seven to twelve years so that he can keep well. The mature critic and especially one expert in physiology, bacteriology and allied sciences will find much that will amuse him, and much with which he will not entirely agree. So far as the grown man can put himself in the place of the little child, it seems to us that the authors have succeeded admirably in preparing text books that will interest and instruct such a child and that will give him or her as correct an idea of the various subjects treated as it is possible to convey to an immature brain. So far as we can judge, most adults underestimate the intelligence of children, do not realize that their craving for information might well be satisfied with systematic instruction along various scientific lines, and that their energy may be directed into channels not only useful to themselves but to the community generally.

---

MANUAL OF SURGERY, by Francis T. Stewart, Professor of Clinical Surgery, Jefferson Medical College. Third edition, 1913. P. Blakiston's, Son & Co.

This book of over 700 pages and 571 illustrations seems to be intended for the undergraduate very largely. It also has its value for that practitioner who wishes quickly to inform himself in a given subject. The book necessarily is not sufficiently comprehensive to give him a complete exposition of a subject, nevertheless, it has a value.

THE SURGICAL CLINICS OF JOHN B. MURPHY, M. D., at Mercy Hospital, Chicago. Volume II, Number V (October, 1913). Octavo of 174 pages, 52 illustrations. Philadelphia and London: W. B. Saunders Company, 1913. Published bi-monthly. Price, per year, paper, $8.00; cloth, $12.00.

The usual variety of operations are detailed. The method of taking down the remarks of an operation is not ideally successful in a precise technical operation of given steps, such as the hernia operation. Fortunately this clinic is amplified by a carefully prepared description and drawings of the Andrew's operation. The case histories are always good, being concise but very much to the point.

